# The SlHB8 Acts as a Negative Regulator in Stem Development and Lignin Biosynthesis

**DOI:** 10.3390/ijms222413343

**Published:** 2021-12-12

**Authors:** Xiaojuan Liu, Caiyu Wu, Deding Su, Yang Yang, Zhiqiang Xian, Canye Yu, Zhengguo Li, Yanwei Hao, Riyuan Chen

**Affiliations:** 1 Key Laboratory of Biology and Genetic Improvement of Horticultural Crops (South China), Ministry of Agriculture and Rural Affairs, College of Horticulture, South China Agricultural University, Guangzhou 510642, China; liuxjjy628@stu.scau.edu.cn (X.L.); wucaiyu1995@163.com (C.W.); 13544392135@163.com (Y.Y.); cyyu@stu.scau.edu.cn (C.Y.); 2 Key Laboratory of Plant Hormones and Development Regulation of Chongqing, School of Life Sciences, Chongqing University, Chongqing 400044, China; dedingsu@163.com (D.S.); tmdxzq@126.com (Z.X.); zhengguoli@cqu.edu.cn (Z.L.); 3 Center of Plant Functional Genomics, Institute of Advanced Interdisciplinary Studies, Chongqing University, Chongqing 400044, China

**Keywords:** SlHB8, tomato, stem development, xylem, lignin

## Abstract

The stem is an important organ in supporting plant body, transporting nutrients and communicating signals for plant growing. However, studies on the regulation of stem development in tomato are rather limited. In our study, we demonstrated that SlHB8 negatively regulated tomato stem development. SlHB8 belongs to homeo domain-leucine zipper Class III gene family transcription factors and expressed in all the organs examined including root, stem, leaves, flower, and fruit. Among these tissues, *SlHB8* showed stable high expression level during tomato stem development. Overexpression of *SlHB8* gene decreased stem diameter with inhibited xylem width and xylem cell layers, while loss of function of *SlHB8* *gene* increased the stem diameter and xylem width. The contents of lignin were decreased both in leaves and stems of *SlHB8* overexpression plants. RNA-seq analysis on the stems of wild type and *SlHB8* transgenic plants showed that the 116 DEGs (differential expressed genes) with reversible expression profiles in SlHB8-ox and SlHB8-cr plants were significantly enriched in the phenylpropanoid biosynthesis pathway and plant-pathogen pathway which were related to lignin biosynthesis and disease resistance. Meanwhile, the key genes involved in the lignin biosynthesis pathway such as *SlCCR* (cinnamoyl-CoA reductase), *SlCYP73A14*/*C4H* (cinnamate 4-hydroxylase), *SlC3H* (coumarate 3-hydroxylase) and *SlCAD* (cinnamoyl alcohol dehydrogenase) were down-regulated in both stem and leaves of *SlHB8* overexpression plants, indicating a negative regulatory role of SlHB8 in the lignin biosynthesis and stem development.

## 1. Introduction

Stems are the central part of the plant, connected with the leaves up and the roots down, and transport important substances for long-distance cell-to-cell communication. Besides, the stem is involved in carbon storage and remobilization of plants, influencing the control of plant’s carbon metabolism [1,2,3]. Therefore, understanding the regulation mechanism of stem differentiation is instrumental. The stem development is moderated by an elaborated regulation network which has been well elucidated in *Arabidopsis* and *woody species* [4,5]. The homeo domain-leucine zipper Class III gene family transcription factors (HD-Zip III) were regarded as one of the key factors during the stem development from stem primary establishment to lateral growth [5].

In *Arabidopsis*, five Class III HD-Zip transcription factors (*REVOLUTA/IFL1* (*REV*), (*PHABULOSA/AtHB14*) *PHB*, *PHAVOLUTA/AtHB9* (*PHV*), *CORONA* (*CAN*/*ATHB-15*), and *ATHB-8*) were isolated with four recognizable domains including a DNA binding homeodomain followed immediately by a leucine zipper motif (HD-Zip); a sterol/lipid binding (START) domain for binding small hydrophobic molecules such as steroid, phospholipids, or carotenoids; and a PAS (Per-ARNT-Sim) domain for protein-protein interaction [6,7]. These five HD-Zip III transcription factors were reported to play roles in the regulation of primary and secondary vascular cell differentiation [8,9,10,11], meristem maintenance [7], leaf patterning [12] and so on. Tortuous stems and leaves, dwarfism, and shortened internodes were found in these genes’ mutants [8,9,10,11,13]. All these five members affect vascular development in *Arabidopsis* by altering their expression levels in a dependent or redundantly way [6]. Overexpression of *ATHB-8* promotes vascular cell differentiation and xylem tissue production in the inflorescence stems of *Arabidopsis* [14], while REV together with PHB and PHV regulated the meristem development in lateral organs [6]. REV, PHB and PHV were revealed to be an activator, while CAN and ATHB-8 were repressors for the formation of interfascicular cambium of the inflorescence stem [6,14]. The expression of HD-Zip III genes was mediated by multiple molecular mechanisms. Such as the small Zip protein (ZPRs) and MiR165/166 [15,16,17,18]. It was reported that ZPR3 inhibited the HD-Zip III protein activity by interacting with HD-Zip III protein to form nonfunctional heterodimers [16,18]. There were *MiR165/166* target sites in the coding sequences of HD-Zip III genes and their expression levels were negatively regulated by *MiR165/166* [15,17].

The lignin content is always related to the stem development and genes affecting stem development also impact lignin biosynthesis [6,19,20,21,22]. Previous studies have identified that members of homeodomain-leucine zipper gene family play important roles in stem tissue development as well as lignin regulation of plants [6,19,20]. For example, knocking down of the *POPCORONA* gene, one member of Class III HD-Zip transcription factor family in populous, results in abnormal lignification in pith cells [9]. *PtoHB7* and *PtoHB8*, the polar HD-Zip III genes, were downstream targets of poplar IAA9-ARF5 module which regulated the secondary growth of poplar woody stems [19]. In *Arabidopsis*, members of the HD-Zip III gene family function differently, the interfascicular fiber of *rev-6* mutant disappeared and lignin decreased, while loss of function of *CNA* gene impacted vascular bundle development and increased lignin content [6]. Ectopic expression of *Zinnia HB12* in *Arabidopsis* regulated xylem parenchyma cells differentiation and up-regulated the expression of genes related to lignin monomer synthesis [20].

Lignin is one of the complex phenylpropanoid polymer, which is one of the main substances in secondary cell walls of plant vascular systems [23]. lignin which widely existed in stem vascular system provided the strength that allows the stem to grow upright [23,24]. Previous research has revealed that lignin is connected to plants’ response to stress [25]. Lignin biosynthesis is affected by the abiotic stress such as drought stress [26], cold stress [27], salt stress [28], nutrient stress such as nitrogen deficiency [29,30,31], calcium deficiency [32], gases stress (CO_2_ and ozone) [33,34], and heavy metals stress [35,36]. Inducing the lignin content or altering the lignin composition enhanced their resistant ability to these abiotic stresses. Such as: In grapevine, overexpression of *VlbZIP30* enhances drought tolerance by activating the expression of lignin biosynthetic genes and increasing lignin deposition [37]. Overexpression of *PaSOD* and/or *RaAPX* in *Arabidopsis* improved plant’s tolerance to salt and cold stress by up-regulation of lignin induced by peroxide [38]. And research on sweet potatoes has found that *IbLEA14* overexpression plants exhibited increased drought and salt resistance due to the increase of lignin content caused by increased expression level of lignin biosynthesis gene [28]. Over expression of two *CBFs* changed the frost sensitivity of Eucalyptus by inducing lignin content and syringyl/guaiacyl (S/G) ratio as well as genes involved in the phenylpropanoid and lignin branch pathway [39]. For the nitrogen fertilization affection on lignin is different with type and tissues examined. In pine (*P. palustris*) seedlings, high-N fertilization reduced the lignin content in roots but had no effect on the lignin in aerial parts of the plant [40]. In populous plants lignin content was increased by high-N due to elevated PAL activity [30]. Apart from abiotic stress, lignin is involved in plant response to biotic stress. Lignin possesses antimicrobial properties that protect plants against pathogenic bacteria [41]. Lignification is induced in response to attack by pathogen including bacteria, fungi and virus [25]. In cotton, suppression of *GhUMC1* reduced lignin biosynthesis genes due to decreased lignin content and further decreased the resistance of plants to *Verticillium.* William has reported that *AtMYB15* transcription factor acted in defense-induced lignification, having the capability of driving lignification, plants of *myb15* mutant showed greater resistance to the bacterial pathogen *Pseudomonas syringae* [42,43,44]. Moreover, lignin can be degraded to chemicals and fuels for industrial applications by many different species of microorganisms including fungi and bacteria, so lignin also protects the structural polysaccharides in plants, from microbial enzyme-mediated hydrolysis [45,46,47]. Besides, lignin is important for the soil carbon cycling. Altering the lignin content in soil affects the bacterial community diversity index [47,48].

Up to now, a total of six HD-Zip III gene family members have been identified in the tomato genome, and *SlHB8* (Solyc08g066500) is one of this family members. To investigate the function of *SlHB8* gene in regulating stem development, *SlHB8* overexpression and *SlHB8* gene knockout lines were generated in this study, of which *SlHB8* was highly expressed and loss of function in stems compared with wildtype, respectively. The transgenic plants carrying SlHB8-ox showed weaker stem and inhibited lignin content, while *SlHB8* gene knocking out lines promoted xylem development but did not impact the lignin content. Moreover, our results revealed that lignin deposition and key genes involved in the lignin biosynthesis pathway were down-regulated both in the leaves and stems of SlHB8-ox lines. These results indicated that the *SlHB8* gene is an essential regulator in stem development and acts as a negative regulator in lignin biosynthesis in tomato.

## 2. Results

### 2.1. SlHB8 Displayed Stable and High Expression Level during Tomato Stem Development

**Figure 1:**
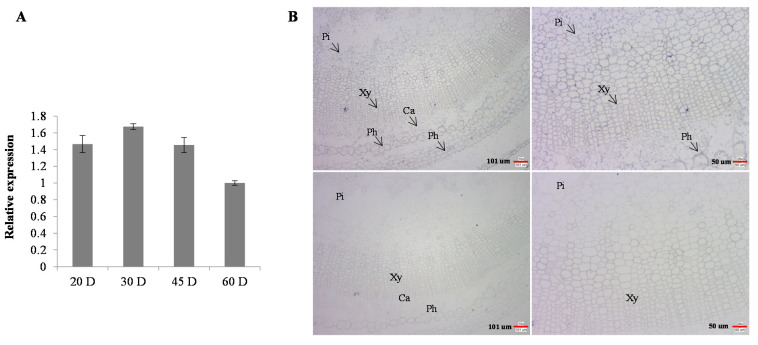
Expression patterns of the *SlHB8* gene in tomato stems. (**A**) Quantitative reverse transcription PCR analysis of the *SlHB8* gene in different development stages of tomato stem. 20 D: 20 days after germination; Error bars mean ± standard error (SE) of three biological replicates. (**B**) RNA in situ hybridization of *SlHB8* in stem tissues of *SlHB8* overexpression tomato plant. Stems at 6th internodes of 2-month-old tomato plants cultivated in soil were cross-sectioned for hybridization with sense (upper) and antisense (lower) probes of *SlHB8*. The photos were taken under 10× (left) and 20× (right) microscopy. Black arrows indicate in situ hybridization signals for *SlHB8* transcripts. Pi, pith; Ca, cambium; Ph, phloem; Xy, xylem. Bars: 101 um (left), 50 um (right).

Previous study showed that *SlHB8* gene belongs to the HD-Zip III transcription factor family, as it contains the four conserved domains of HD, bZip, START and MEKHLA in the HD-Zip III transcription factor [49]. Meanwhile, it expresses in all the tissues such as: root, stem, leaves, flower, mature green fruits, breaker fruits and red fruits and shows the highest expression level in stem tissue [49]. To understand the possible function of the *SlHB8* gene in tomato stem development, we checked its expression pattern in stems at different developmental stages by quantitative reverse transcription-polymerase chain reaction (qRT-PCR). The results showed that *SlHB8* gene expressed in all the stages examined, including 20 D, 30 D, 45 D and 60 D stages stem tissues. Among these stages, the relative transcript level of *SlHB8* gene maintained stable high in tomato stems at 20 D, 30 D, and 45 D stages but decreased a little in tomato stem at 60 D stage (Figure 1A).

### 2.2. SlHB8 Affects Tomato Stem Development through Mediating the Xylem Range

**Figure 2:**
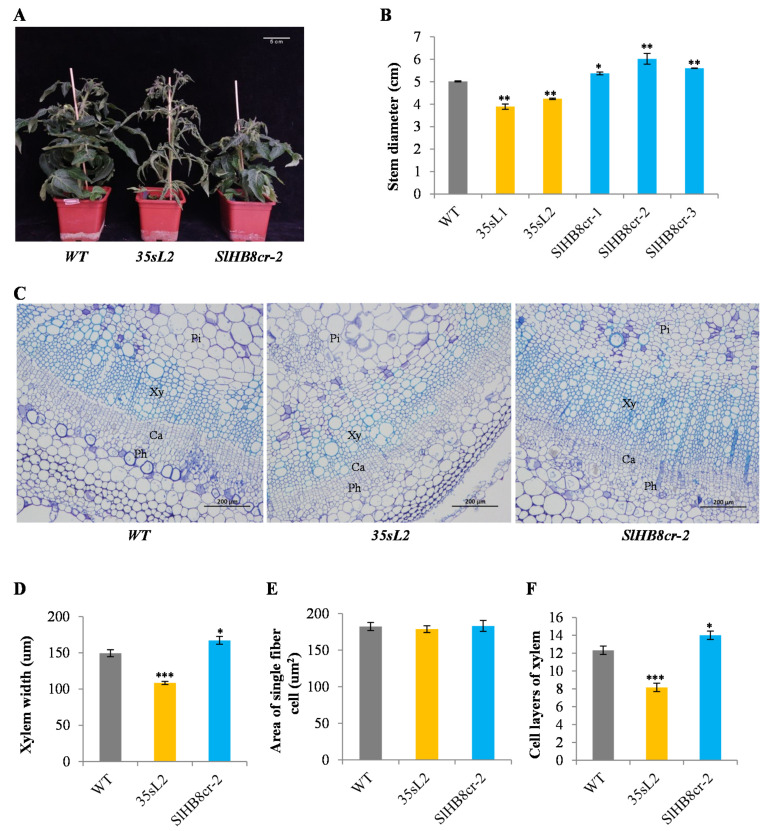
Phenotype analysis of *SlHB8* overexpression and *SlHB8* knock out lines. (**A**) Photos of adult plants of representative two-month-old *SlHB8* overexpression and *SlHB8* knock out lines. Bar: 5 cm; (**C**) Cross-sectioning and staining with toluidine blue of the 6th internode of 2-month-old wild-type, *SlHB8* overexpression and *SlHB8* knock out lines. Pi, pith; Ca, cambium; Ph, phloem; Xy, xylem. Bars: 200 um; (**B**) Measurement of stem diameter, (**D**) xylem width, (**E**) a single fiber cell size and (**F**) xylem cell layers in *SlHB8* overexpression and *SlHB8* knock out lines as well as WT plants. The calculation was performed on IMAGE J softer ware based on the images of toluidine blue-stained anatomical sections as described in the Materials and Methods section. In the bar chart, the gray barplots represent the wildtype line, the orange barplots represent the 35s-driven *SlHB8* overexpression line, and the blue barplots represent the *SlHB8* knockout line. Error bars mean ± standard error (SE) value. Stars indicate the statistical significance using Student’s *t*-test: * *p*-value < 0.05, ** *p*-value < 0.01, *** *p*-value < 0.001.

To identify its role in regulating stem development, we generated *SlHB8* gene knockout mutant by using CRISPR/Cas9 technology (Figure S1A) and *SlHB8* overexpressed transgenic tomato lines (Figure S1B). Three kinds of *SlHB8* loss of function mutants were verified by sequencing the sgRNA target site (Figure S1A). Expression analysis by qRT-PCR showed that the relative transcript level of *SlHB8* was strikingly upregulated in overexpression of *SlHB8* lines (35sL1; 35sL2) (Figure S1B–D) but was specifically reduced in SlHB8-cr lines compared with wild type, respectively (Figure S1C,D). Comparing to wild type plant, overexpression of the *SlHB8* gene did not change plant height and internode length of stem, while loss of function of *SlHB8* gene led to a 14 % reduction in plant height and the reduced plant height resulted from a 15 % reduction in internode length (Figure 2A and Figure S1E,F). Increasing the relative transcription level of the *SlHB8* gene or knock out of *SlHB8* gene did not change the number of nodes in the plant (Figure S1G). Phenotypic observation on stem diameters revealed that compared to wild type plant, overexpressing *SlHB8* reduced stem diameter while loss of function of *SlHB8* gene increased stem diameter (Figure 2C). To further understand the changed stem diameters in *SlHB8* transgenic plant, we examined phenotypes of stem-associated cell types by carrying out the paraffin section analysis in WT and *SlHB8* transgenic plants. There were apparent differences in the range of xylem in stems among different lines. These xylem cells were stained by toluidine blue. Quantitative measurement showed that compared with the wild type, overexpression of the *SlHB8* gene reduced the xylem width of the tomato stem, while the xylem width enlarged in SlHB8-cr lines (Figure 2B,D and Figure S2A). Overexpressing *SlHB8* repressed the xylem development, with a 34 % decrease in the number of xylem cell layers, but *SlHB8* gene knocking out increased the number of xylem cell layers by 12 %, compared with WT (Figure 2D,F). Furthermore, we measured the single cell size of xylem fibers, which had no obvious difference in all genotype plants (Figure 2E). The characteristics of pitch cells examination showed that overexpression of *SlHB8* reduced the area of individual pitch cells in the stem and knocking out of *SlHB8* gene did not result in significant differences in the size and number of pitch cells compared with WT (Figure S2B). Interestingly, compared to the wild type, the size of xylem vessel cells did not change in the SlHB8-ox lines but decreased in SlHB8-cr mutants (Figure S2C). To clarify whether the changed xylem width is related to the expression level of *SlHB8*, we determined the expression position of *SlHB8* in the SlHB8-ox lines by using the RNA in situ hybridization on stems at the sixth internode of 2-month-old tomato (Figure 1B). The results revealed that strong expression signals of *SlHB8* positive probes were observed in the area of pith, xylem, phloem and cambium regions compared with those of negative probes (Figure 1B), suggesting that *SlHB8* gene was overexpressed in these tissues. Collectively, these data indicated that *SlHB8* affects stem diameter by mediating the xylem range.

### 2.3. SlHB8 Affects Lignification in Tomato Stems and Leaves

**Figure 3:**
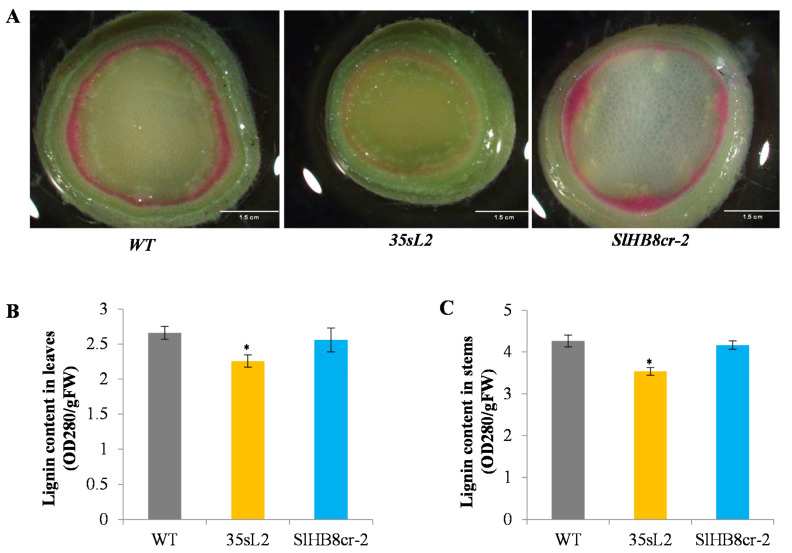
*SlHB8* affects lignification in tomato leaves and stems. (**A**) Free-hand sections of the 2-month-old stem were subjected to phloroglucinol-HCl staining. The red area represents lignin. Bars: 1.5 cm. (**B**) Acetyl bromide-soluble lignin assays were carried out on leaves (2-month-old tomato) of *SlHB8* overexpression, *SlHB8* knock out lines and WT plants. (**C**) The content of lignin in the stems (2-month-old tomato) of *SlHB8* overexpression, *SlHB8* knock out lines and WT plants was measured by acetyl bromide lignin assay. In the chart of B and C, the gray columns represent the wildtype line, the orange columns represent the 35s-driven *SlHB8* overexpression (35sL2) line, and the blue columns represent the *SlHB8* knockout (SlHB8-cr2) line Error bars mean ± standard error (SE) value for each line. Stars indicate the statistical significance using Student’s *t*-test: * *p*-value < 0.05.

As stem diameter is always positively related to the lignin biosynthesis, we examined the lignin content in stem tissues of *SlHB8* transgenic plants by histochemical staining with hydrochloric acid-phloroglucinol which is used for lignin staining analysis. Staining results showed that compared with WT, the xylem of SlHB8-ox had a small lignin deposition area and significantly reduced staining brightness, indicating a decrease in lignin content, however, there was no significant difference in lignin deposition between SlHB8-cr and WT (Figure 3A). To confirm the level of lignification, the total lignin content in WT and *SlHB8* transgenic plants was measured by the acetyl bromide (AcBr) method. Consistent with staining analysis result, the lignin content significantly decreased in stems and leaves of SlHB8-ox lines while not changed in stems and leaves of SlHB8-cr plants (Figure 3B,C).

### 2.4. Transcriptomic Analysis of WT, SlHB8-ox and SlHB8-cr Plants

**Figure 4:**
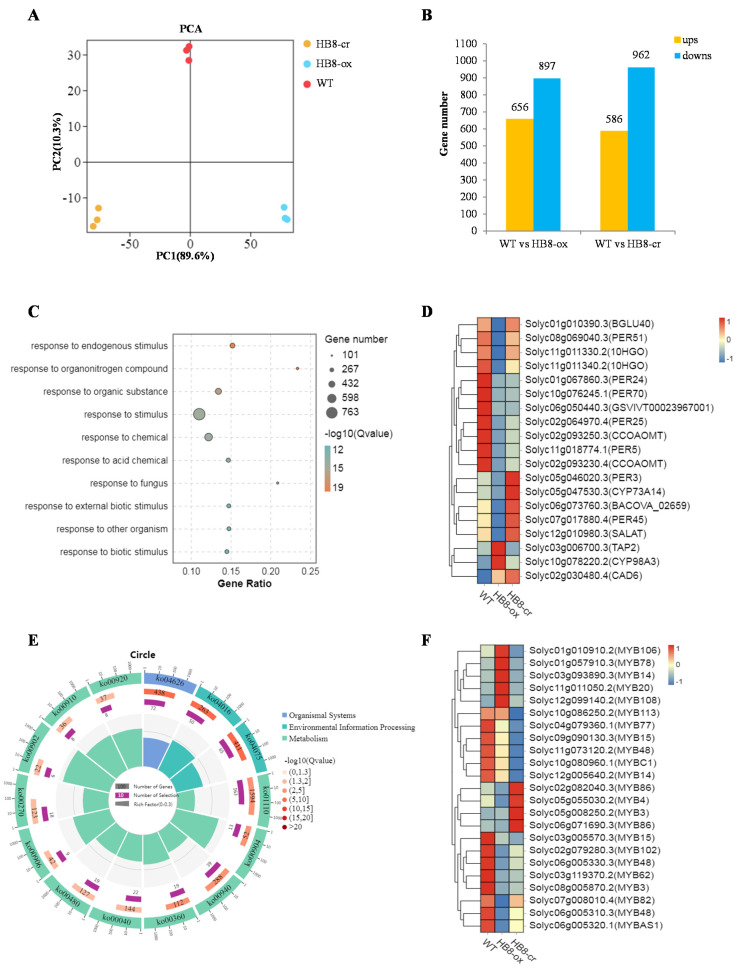
Differentially expressed genes (DEGs) analysis in WT, SlHB8-ox, and SlHB8-cr plants. (**A**) Principal component analysis (PCA) of the three group samples (WT, red; SlHB8-cr, yellow; SlHB8-ox, blue); the x-axis represents the first principal component and the y-axis represents the second. (**B**) Histograms showing the DEGs number in WT vs. SlHB8-ox and WT vs. SlHB8-cr. (**C**) Top ten significantly enriched GO terms. (**D**) Heatmap of DEGs involved in the phenylpropanoid biosynthesis pathway. (**E**) Heatmap of DEGs belong to the MYB transcription factor. (**F**) Significantly enriched KEGG terms.

To better understand the molecular mechanism of *SlHB8* regulation of stem development, RNA-seq was carried out on stems of 2-month-old plant of WT, SlHB8-ox, and SlHB-cr mutant. Three biological replicates were included in each sample and finally generated 9 libraries. The high-quality clean reads of the library reached over 99 % (Table S1). After filtering the rRNA, the library was uniquely mapped to the tomato genome (*Solanum lycopersicum* ITAG4.0). The mapped reads ranged between 97.04 % and 97.46 % and unique mapped reads ranged from 94.61 % to 95.32 % (Table S1). The annotated gene numbers in the 9 libraries ranged from 22,265 to 22,885. A total of 627 novel transcripts were identified from the 9 libraries, each containing more than 570 novel genes (Table S1). Principal component analysis (PCA) of the RNA-seq samples revealed highly repeatability between three replicates of each sample of the wild type, SlHB8-ox, and SlHB8-cr, and great differences among the stems of 2-month-old tomato in different lines (Figure 4A). The RNA-seq analysis showed a 3.8-fold difference in *SlHB8* expression between wild-type and SlHB8-ox stems (*p* < 0.001, Student’s *t*-test), and the expression of the *SlHB8* gene in the SlHB8-cr stems was 0.29 times than that in the, wild type stems (*p* < 0.001, Student’s *t*-test) closely corresponding to the results obtained by real-time quantitative PCR analysis (Figure S1C,D).

To identify candidate genes that are vital for stem development, we performed a comprehensive analysis of gene expression in stems at the 6th node of the 2-month-old tomato of WT, SlHB8-ox, and SlHB-cr mutant. Genes that satisfied the fold-change difference |log2 (fold-change)| > 1 and FDR < 0.05 were regarded as differentially expressed genes (DEGs). 1553 (656 up-regulated + 897 down-regulated) DEGs were detected in the comparison between WT and SlHB8-ox plants, and 1548 (586 up-regulated + 962 down-regulated) DEGs were found in the comparison between WT and SlHB8-cr plants (Figure 4B; Table S2). A total of 2592 DEGs were found between WT and *SlHB8* transgenic plants (Table S2). To gain further insight into the putative functions of these DEGs between the wild type and *SlHB8* transgenic lines, GO assignment and Kyoto Encyclopedia of Genes and Genomes (KEGG) database were used for the further analysis. Using q value ≤ 0.05 as the significant cut-off, the data revealed that these 2592 DEGs were significantly enriched in the GO terms related to disease resistance such as “response to endogenous stimulus,” “response to stimulus,” “response to fungus,” “response to external biotic stimulus” and “response to biotic stimulus”(Figure 4C; Table S3) and 14 KEGG pathways were significantly enriched (Figure 4D; Table S3) including pathways related to disease resistance and lignin biosynthesis such as “plant-pathogen interaction,” “MAPK (mitogen-activated protein kinase) signaling pathway-plant” and “phenylpropanoid biosynthesis”. As the lignin content was reduced in the *SlHB8* overexpressing plant, we further analyzed the expression profile of DEGs related to lignin biosynthesis. The heatmaps revealed there were 19 DEGs differently expressed in the *SlHB8* transgenic plant with 16 down-regulated in the SlHB8-ox lines which may account for the decreased lignin content (Figure 4F; Table S2). 23 *MYBs* were found differently expressed in the *SlHB8* transgenic plant including 13 down-regulated and 4 up-regulated in the SlHB8-ox lines (Figure 4E; Table S2). All of these suggested that *SlHB8* gene might regulate the synthesis of lignin.

**Figure 5:**
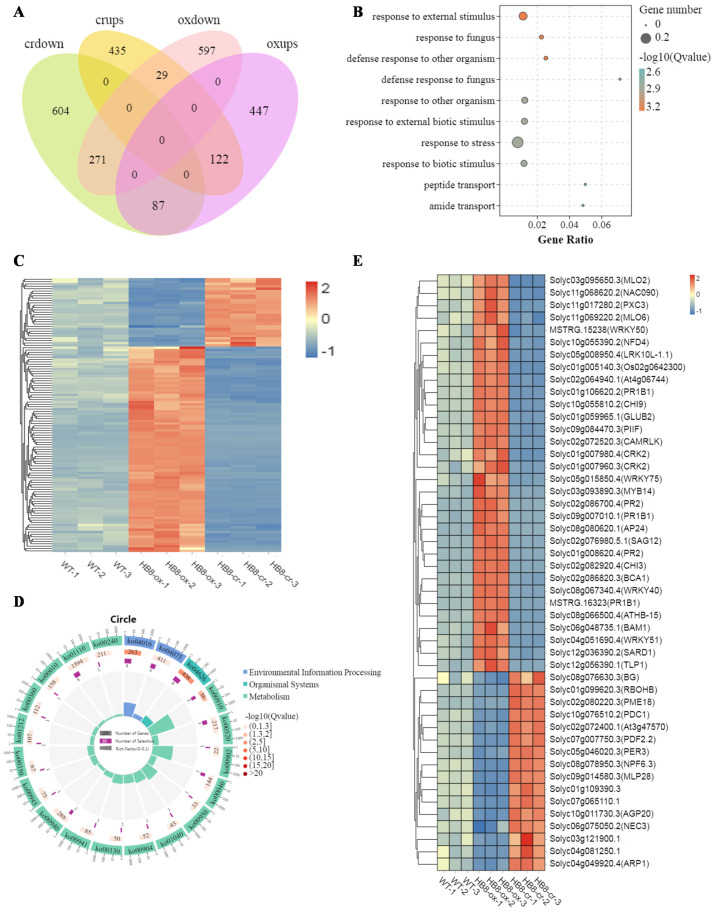
Prediction and functional analysis of DEGs directly regulated by SlHB8. (**A**) Venn diagrams selecting 116 DEGs with reversible expression pattern potentially directly regulated by SlHB8. (**C**) Heat maps of predicted 116 DEGs directly regulated by SlHB8. (**B**) Significantly enriched GO terms based on the 116 DEGs; (**D**) Significantly enriched KEGG terms based on the 116 DEGs; (**E**) Heatmap of DEGs related to disease resistance.

Aims to narrow the range of SlHB8 regulated genes, genes with reversible expression profiles in *SlHB8* overexpression and *SlHB8* knock out lines were selected by overlapping the differentially expressed DEGs gene sets. The Venn diagram revealed that there were 116 DEGs with reversible expression pattern including 29 DEGs up-regulated in SlHB8-cr and down-regulated in SlHB8-ox and 87 DEGs down-regulated in SlHB8-ox and up-regulated in SlHB8-cr (Figure 5A,C; Table S2). GO and KEGG functional analysis displayed these 116 DEGs were enriched in the GO terms of response to fungus, response to biotic stimulus, immune system process, and salicylic acid mediated signaling pathway which acts in the disease response pathway (Figure 5B; Table S3); in the KEGG pathways of MAPK signaling pathway and plant-pathogen interaction (Figure 5D; Table S3), indicating SlHB8’s role in the disease resistance. 47 out of 116 genes were related to disease resistance, among which 31 were down-regulated in SlHB8-ox and up-regulated in SlHB8-cr (Figure 5E; Table S2).

### 2.5. SlHB8 Alters the Expression Level of Several Genes Related to Lignin Biosynthesis in Leaves

**Figure 6:**
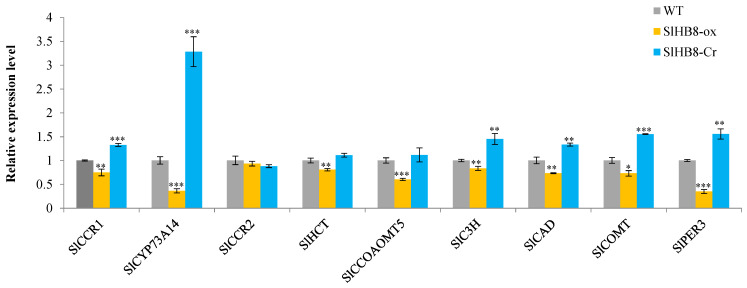
SlHB8 regulates the expression of the phenylpropanoid biosynthesis pathway. The expression pattern of key genes of lignin synthesis pathway in the leaves of WT, SlHB8-ox and SlHB8-cr plants were analyzed by RT-qPCR. In the chart, the gray columns represent the wildtype line, the orange columns represent the 35S-driven SlHB8 overexpression (35sL2) line, and the blue columns represent the *SlHB8* knockout (SlHB8-cr2) line. Error bars mean ± standard deviant [SE] and stars indicate the statistical significance. Student’s *t*-test: * *p*-value < 0.05, ** *p*-value < 0.01, *** *p*-value < 0.001.

As lignin content was also reduced in leaves of SlHB8-ox lines, we checked the expression level of genes involved in the lignin biosynthesis pathway by using RT-qPCR. The result showed that *SlCCR1* (cinnamoyl-CoA reductase), *SlCYP73A14/C4H* (cinnamate 4-hydroxylase), *SlCAD* (cinnamoyl alcohol dehydrogenase), *SlC3H* (coumarate 3-hydroxylase), *SlCOMT* (caffeic acid-3-O-methyltransferase) and *SlPER3* (peroxidase 3 precursor) were down-regulated in SlHB8-ox lines and up-regulated in SlHB8-cr lines. *SlHCT/C3H* and *SlCCOAOMT5* (caffeoyl-CoA 3-O-methyltransferase) were only reduced in the SlHB8-ox lines. The expression level of *SlCCR2* was not changed. All of these genes’ expression profiles were consistent with that in stem (Figure 6). Among the 116 genes, there were two genes’ promoters containing SlHB8 binding site, of which peroxidase 3 precursor (*PER3*) and cytochrome P450 *CYP73A14*(*C4H*) were involved in lignin biosynthesis (Table S6), indicating a direct regulation between SlHB8 and these two gene.

### 2.6. Validation of RNA-Seq Data by Quantitative Real-Time Polymerase Chain Reaction (qRT-PCR)

To investigate the accuracy and reproducibility of the RNA-seq data, 15 DEGs were selected from RNA-seq results for qRT-PCR (Table S4). We amplified 15 genes by qRT-PCR using specific primers to confirm the accuracy and reproducibility of RNA-seq expression profiles. The results revealed that all 15 genes displayed the same trend (Figure S3), implying that the RNA-seq was reliable.

## 3. Discussion

### 3.1. SlHB8, as a Negative Regulator, Affects Tomato Stem Development by Mediating Xylem Range

Despite significant progress towards understanding the roles of HD-Zip III family genes in vascular development in many species [2], our knowledge about its role in the tomato stem development is still in infancy. Indeed, except for a recent report showing that SD1, a kinase-interacting family protein positively regulates stem diameter by controlling the size and number of secondary phloem cells [1], no data are presently available about the potential roles of HD-Zip III genes in stem development in tomato. In our study, we isolated one of the HD-Zip III family gene *SlHB8* from tomato and proved its negative role in the xylem development.

First, the expression pattern result revealed that *SlHB8* expression level was related to stem development with steady higher transcripts accumulation during stem development. The in-suit hybridization result showed *SlHB8* expressed in the xylem tissues of the stem. Second, overexpression of *SlHB8* under 35s promoters reduced stem diameter and xylem range. Loss of function of *SlHB8* by using CRISPR/Cas 9 assay promoted stem and xylem development. Overall, we hypothesized that SlHB8 as a negative regulator regulates xylem development during stem formation.

In *Arabidopsis*, *AtHB8* displayed specific expression in procambial cells and its role in xylem development [14]. Overexpression of *AtHB8* enlarged the xylem tissues [14], which is different from the *SlHB8* in tomato. The *AtHB8* homolog gene *PtHB7* in populous was also expressed in the xylem tissues and overexpression of *PtHB7* decreased the xylem distance in the populous stem, which is consistent with the role of *SlHB8* in tomato [10]. Both in *Arabidopsis* and *Populous*, there is auxin binding site in the promoters of *AtHB8* and *PtHB7*, and ARF5 binds this site. The conserved pathway of ARF5-HB7/8 for vascular patterning of leaves and stems was found in the herbaceous and woody species [19,50]. We also found auxin binding sites in the promoter of *SlHB8*, whether the conserved pathway is also appeared in tomato remains to be clarified.

### 3.2. SlHB8 Negatively Regulates Lignin Biosynthesis in Tomato Leaves and Stems

The *HB8* gene was reported to function in the xylem development and leaf patterning [14,51,52,53,54], whereas its role in the lignin biosynthesis was not yet clarified. In our study, lignin content was reduced in both leaves and stems of *SlHB8* overexpression lines (Figure 3), moreover, the down-regulated DEGs in *SlHB8* overexpression lines were significantly enriched in the phenylpropanoid biosynthesis pathway which generates lignin polymers, indicating its role in the lignin formation. 19 DEGs in the phenylpropanoid biosynthesis pathway were found differentially expressed in the *SlHB8* overexpressed lines, 16 out of these 19 DEGs were down-regulated which may account for decreased lignin levels (Figure 4D). Meanwhile, the SlHB8 binding sites were found in the promoter of *SlPER3* which is involved in the Casparian strips’ formation [55].

During recent years, an increasing amount of evidence has indicated that phenylpropanoid biosynthetic genes may involve the combinatorial actions of different transcriptional activators and repressors, and R2R3-MYB transcription factors play important roles in the phenylpropanoid biosynthesis pathway [56]. In our study, 23 *MYBs* were differentially expressed in the *SlHB8* overexpression or knocking out plants. Almost 17 out of 23 *MYBs* were down-regulated in the *SlHB8* overexpression lines, which may contribute to the reduced lignin level. The expression level of some *MYB* homolog genes of *Arabidopsis* related to the lignin biosynthesis were altered in the *SlHB8* transgenic plants. The homolog of *AtMYB15* (solyc03g005570) which was reported to be required for the activation of lignin biosynthesis genes such as *PAL* (phenylalanine ammonialyase), *C4H* (cinnamate 4-hydroxylase), *4CL* (coumarate CoA ligase), *HCT*/*C3H* (coumarate 3-hydroxylase), *COMT* (caffeic acid-3-O-methyltransferase), and *CAD* (cinnamoyl alcohol dehydrogenase) [42,43,44] was down-regulated in the *SlHB8* overexpression and knocking out lines. The homolog genes of *AtMYB58* (Solyc03g093890) and *AtMYB61* (Solyc10g044680) which were positively regulated the lignin content by activating *PAL*, *4CL*, *CCR* (cinnamoyl-CoA reductase) and *CAD* [57,58] were up-regulated in the *SlHB8* transgenic plants. The homologs genes of negative regulators of phenylpropanoid biosynthesis pathway such as *MYB3* [59] (Solyc06g065100) and *MYB4* [60] (Solyc10g055410) were also induced in the *SlHB8* overexpressing lines. The SlHB8 binding sites were also found both in these promoters of activators and repressors, indicating a direct regulation between SlHB8 and *SlMYBs* in the lignin biosynthesis pathway. Overall, all these data imply *SlHB8* participates in an elaborate regulation network in the phenylpropanoid biosynthesis pathway.

### 3.3. SlHB8 May Involve in the Disease Resistance

Lignification plays an important role in disease resistance. The lignin biosynthetic genes and disease resistance are positively correlated [61,62]. GO and KEGG functional analysis of the DEGs between wild type and SlHB8 transgenic plant revealed these DEGs were enriched in the plant-pathogen interaction pathway and phenylpropanoid biosynthesis pathway. Moreover, the lignin content together with phenylpropanoid biosynthesis pathway genes were both reduced in the *SlHB8* overexpression lines indicating the reduced disease resistance in the *SlHB8* overexpression plants. Besides, genes regulating the lignin biosynthesis and pathogen resistance were also found differentially expressed in the *SlHB8* transgenic plants. *AtMYB15* (solyc03g005570) is a regulator of defense-induced lignification and basal immunity and loss of function of *AtMYB15* reduced lignin deposition and resistance to a virulent bacterial pathogen *Pst* DC3000 [42,43,44]. The homolog gene of *AtMYB15* (solyc03g005570) was reduced in the *SlHB8* transgenic plants. *CASPLs* determine lignin accumulation in the Casparian strip which is mechanical barrier to prevent the spread of pathogens [63]. The homolog gene of *CASPLs* (Solyc01g067300) showed an opposite expression level in *SlHB8* overexpressing and knockout lines. In addition, 116 DEGs predicted to be directly regulated by SlHB8 were enriched: in the GO terms of response to fungus, response to biotic stimulus, immune system process, and salicylic acid mediated signaling pathway which acts in the disease response pathway; in the KEGG pathways of MAPK signaling pathway and Plant-pathogen interaction, further indicating SlHB8′s direct regulating role in the disease resistance. Among these 116 genes there were plenty of genes related to the disease resistance such as: the homolog genes of *FLS2* (solyc02g072400, solyc06g048735) which is the recognition receptor of *flag22* who triggered plant immune response on pathogen attack [64,65]; the homolog genes of pathogenesis-related genes (*PRs*) (MSTRG.16323, solyc01g106620, solyc09g007010) which were reported to be induced and determined the disease resistance in plants [66,67,68]; the homolog genes of *RBOH* (Solyc01g099620) which is positively related to the pathogen resistance to nematodes in leaf-infecting of *Arabidopsis* [69]. In addition, the homolog genes of *CRK2* (Solyc01g007960, Solyc01g007980) who formed a complex with RBOHD for the elicitor-induced ROS burst and loss of function of *CRKs* impaired the plant defense against the bacterial pathogen *Pseudomonas syringae* pv *tomato* DC3000 [70]; the homolog gene of *MLO2* (Solyc03g095650) which is called mildew resistant Locus O (MLO) proteins modulating the plant susceptibility to powdery mildew fungi. Loss of function mutant of *mlo2*, *mlo6*, *mol12* and *mlo3* improved the resistance ability [71].

Besides, some transcription factors involved in regulating plant resistance to disease were also found differentially expressed in the *SlHB8* transgenic plants. The basic leucine zipper transcription factors TGA1 and TGA4 regulate SA biosynthesis by modulating the expression of *SARD1* and *CBP60g* to prevent the pathogen infection [72]. WRKY transcription factors have also been shown to regulate cross-talk between JA and SA-regulated disease response pathways. Mutations of the *Arabidopsis WRKY33* caused enhanced susceptibility to the necrotrophic fungal pathogens *Botrytiscinerea* and *Alternaria brassicicola* concomitant with reduced expression of the JA-regulated plant defensin *PDF1.2* gene. The susceptibility of *WRKY33*-overexpressing plants to *P. syringaeis* associated with reduced expression of the salicylate-regulated *PR-1* gene [73]. Overall, *SlHB8* is predicted to be a regulator in the lignin biosynthesis and disease resistance.

In conclusion, the determinant of the natural variation influencing stem diameter in natural populations of tomato is Indel 11 in the promoter of the *SD1* gene [1]. *SD1* is the first domesticated gene related to stem diameter by regulating cell expansion and cell number in parenchyma tissue of stem [1]. Except *SD1*, few molecular regulation of stem development have been reported in tomato. Besides Indel 11, another nine loci that influence stem development were rigorously verified and the further regulation mechanism need to be clarified [1]. In our study, we indicate that SlHB8 negatively regulates stem thickening by mediating the xylem width which is also relevant to the lignin content. The role of *SlHB8* will contribute to the molecular mechanism of stem development in tomato. Whether *SlHB8* can be used for the import loci of the stem development, further research needs to be done.

## 4. Materials and Methods

### 4.1. Plant Materials, Growth Conditions, and Plant Transformation

The overexpression lines of *p35s::SlHB8* were generated by cloning the full length CDS of *SlHB8* (Solyc08g0066500) into plant overexpression vector pMDC32 which was transformed into *Agrobacterium tumefasciens* for tomato genetic transformation. The *SlHB8* knockout mutants generated by using CRISPR/Cas 9 were provided by the lab of Chongqing university. One sgRNA (GCAGAAGCAAGTTTCACAGT) in the coding sequence of *SlHB8* was cloned into the vector pAGM4723 and then used for tomato genetic transformation. Three kinds of *SlHB8* knockout mutant were obtained including two types of 8 bps deletion in the CDS and 1 bp addition in the CDS. Wildtype (*Solanum lycopersicum* L. “Micro-Tom”) and *SlHB8* transgenic plants were grown in a greenhouse at the College of Horticulture of the South China Agriculture University. The environmental conditions of the greenhouse are 25 ± 1 °C with a photoperiod of 16 h/8 h (light/dark).

Stems at 20 D (20 days after germination), 30 D (30 days after germination), 45 D (45 days after germination), and 60 D (60 days after germination) stages were sampled for analysis. The 6th node of the 2-month-old tomato stem samples of each line were immediately frozen in liquid nitrogen and stored at −80 °C until use.

### 4.2. Determination of Characteristics Related to Stem Development

The stem diameter of the 6th internode of the 2-month-old tomato plants was measured by a vernier caliper. The microscopic characteristics related to stem development such as xylem width, area of single cell, cell layers of xylem and area of a signal vessel cell were measured by the Image J software (Image-Pro Plus 6.0) based on the images of toluidine blue-stained paraffin sections.

### 4.3. Paraffin Transverse Section of Stem Tissues

Stem samples were fixed in FAA solution (70 % ethanol: formaldehyde: glacial acetic acid, 18:1:1). After a series of processes such as 50–100 % alcohol gradient dehydration, tissue transparency and paraffin infiltration, the stem tissue are embedded in paraffin. The specimens were cut into thin sections of 8 um, and after dewaxing and rehydration treatment, all sections were stained with 0.5 % toluidine blue. The cross-sections were observed and captured under Zeiss Axio Scope (Zeiss, Oberkochen, Germany).

### 4.4. Phloroglucinol-HCl Staining Analysis

Hand-cut cross sections of 2-month-old WT and two kinds of *SlHB8* transgenic plants stems were stained with 1.0 % (Weight/Volume, *w*/*v*) phloroglucinol, then dissociated by 30 % (Volume/Volume, *v*/*v*) HCl (hydrochloric acid), and finally observed and captured by Bioscope.

### 4.5. Measurement of Lignin Content

Leaves and stems tissue of 2-month-old WT and two kinds of *SlHB8* transgenic plants were used to determine lignin contents. The method of lignin content was previously described by Su et al. [19]. The tissues used for lignin determination were collected from the same part of different plants.

### 4.6. RNA-Seq Analysis

Stem tissues were collected from 2-month-old plants of WT, SlHB8-ox and SlHB8-cr. Each sample contained three biological repeats, and each sample included at least 3 stems. All samples were sent to Guangzhou Gene Denovo Biological Technology Co., Ltd. (Guangzhou, China) for RNA extraction and RNA-Seq library preparation and sequencing. The cDNA libraries were sequenced using the Illumina HiSeqTM 2500. False discovery rate (FDR) < 0.05 control method and an absolute value of |log2 (fold change)| > 1 as the threshold were used to determine the differentially expressed genes (DEGs). Gene ontology (GO) terms and the Kyoto Encyclopedia of Genes and Genomes (KEGG) database were used to further analyzed DEGs enrichment. Transcriptome data analysis and mapping were carried out using OmicShare Tools (www.omicshare.com/tools, accessed on 18 November 2021), a free online platform developed by Guangzhou GENE DENOVO Biotech.

### 4.7. RNA Extraction and Real-Time Quantitative PCR Analysis

The total RNA of tomato leaves was extracted by using a E.Z.N.A. Plant RNA extraction Kit (Omega Bio-tek, Inc., GA, USA), which includes a genomic DNA elimination step. Total RNA from stem samples was provided by Gene Denovo Biological Technology Co., Ltd. (Guangzhou, China). The cDNA was synthesized using the PrimeScript RT Reagent Kit with gDNA Eraser (Takara, Guangzhou, China), according to the manufacturer’s instructions. We selected 15 DEGs from RNA-seq data for RT-qPCR analysis to verify the results of RNA-seq. RT-qPCR was performed in a 10 μL reaction volume containing 5 μL of 2 × TB Green Master Mix Reagent (Takara, Guangzhou, China), 1 μL of cDNAs and 4 μL of gene-specific primers (Table S5), which were designed using Primer-BLAST in National Center for Biotechnology Information (NCBI). The expression levels of housekeeping gene SlUBI was used as reference for calculating the relative expression of target gene using the 2^−∆∆Ct^ method [21]. RT-qPCR analysis was based on three biological replications and three technical replications.

## 5. Conclusions

SlHB8 belongs to the HD-Zip III transcription factor family and shows stable high expression level during tomato stems development. Loss of function of *SlHB8* induced stem diameter and xylem width, while overexpression of *SlHB8* displayed opposite trend. Besides, inducing the expression level of *SlHB8* resulted in lower lignin content as well as the expression level of lignin biosynthesis pathway genes both in tomato stem and leaves. In addition, lots of disease resistance genes were found differentially expressed in the *SlHB8* transgenic plants indicating a possible role of *SlHB8* in the biotic resistance pathway. Overall, SlHB8 acts as a negative regulator in stem development and lignin biosynthesis and has a potential role in the abiotic and biotic resistance pathway.

## Data Availability

All important data is included in the article and Supplementary Materials.

## Supplementary Materials

The following are available online at https://www.mdpi.com/article/10.3390/ijms222413343/s1.
