# Supplementary material for: The SlHB8 Acts as a Negative Regulator in Stem Development and Lignin Biosynthesis
Source: Int J Mol Sci. 2021 Dec 12;22(24):13343. doi: 10.3390/ijms222413343 (PMC8708474; doi:10.3390/ijms222413343)
Supplement: Supplementary file 1 [file ijms-22-13343-s001.zip › ijms-1492812-SI/supplementary figures.pdf]

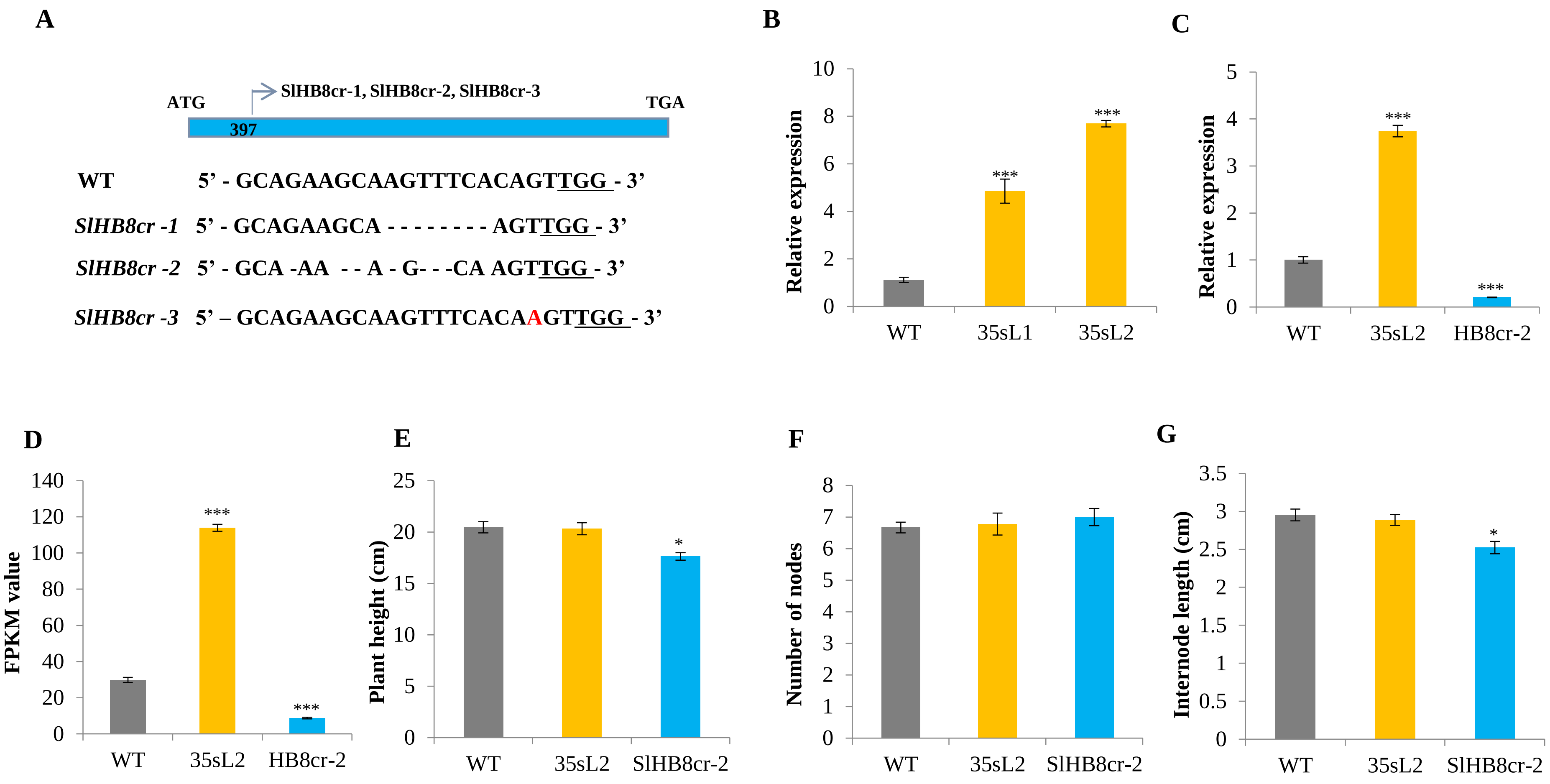

Figure S1. (A) Three kinds of SlHB8 gene editing plants by using CRISPR/Cas 9 system. (B) Quantitative reverse transcription PCR analysis of SlHB8 genes in leaves of SlHB8 overexpression lines as well as wild type plant. (C) Quantitative reverse transcription PCR analysis and RNA-seq analysis (D) of SlHB8 genes in stem of SlHB8 overexpression lines, SlHB8 knock out line and wild type plant. Measurement of the plant height (E), number of nodes (F) and internode length (G) of SlHB8 overexpression, SlHB8 knock out lines and WT plants. Error bars mean ± SE value for each line. Stars indicate the statistical significance using Student's t-test: * p-value < 0.05, ** p-value < 0.01, *** p-value < 0.001.

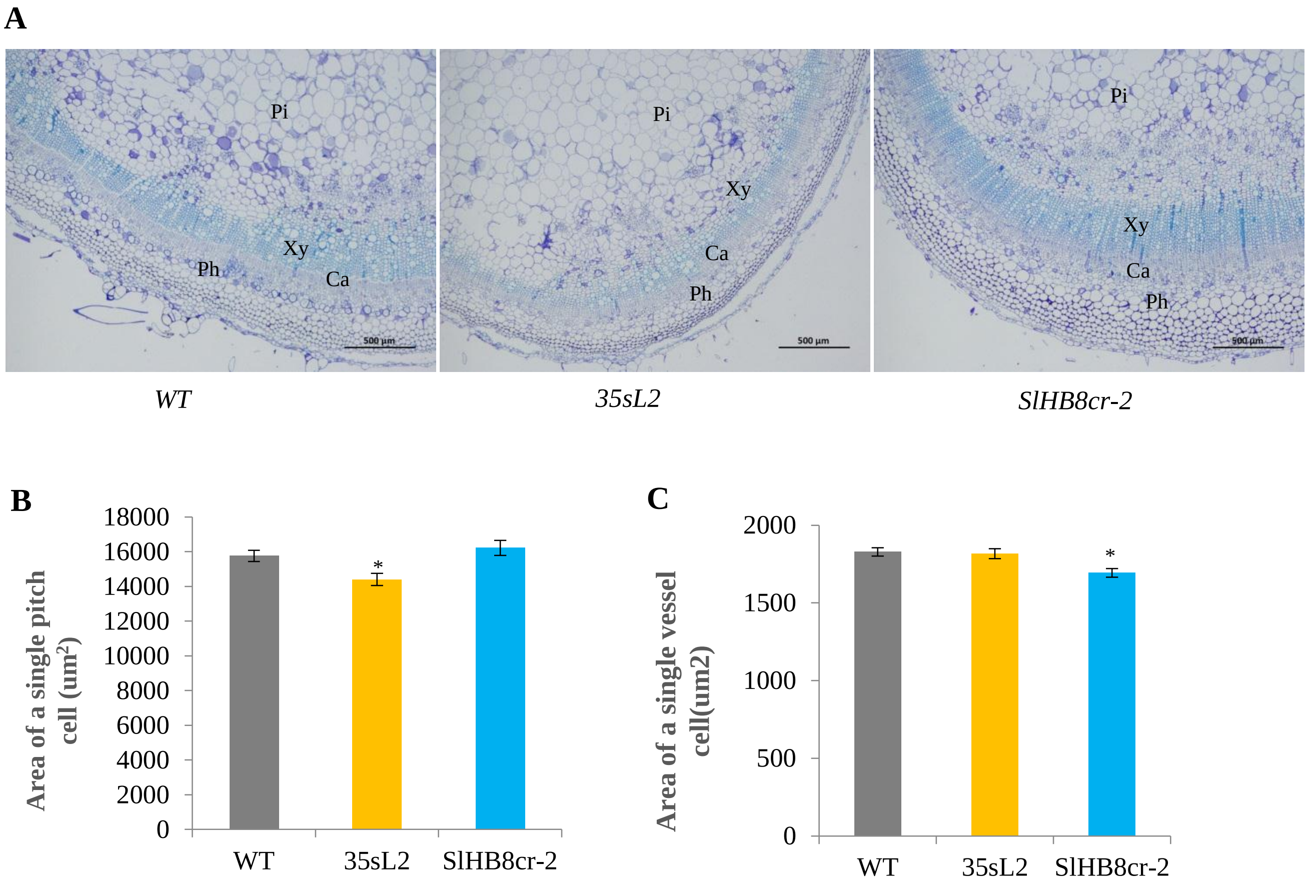

Figure S2. Characteristics of stem cells from paraffin sections of SlHB8 overexpression and SlHB8 knock out lines as well as WT plants. . (A) Cross-sectioning and staining with toluidine blue of the 6th internode of 2-month-old wild-type, SlHB8 overexpression and SlHB8 knock out lines. Pi, pith; Ca, cambium; Ph, phloem; Xy, xylem. Bars: 500um; The area of a single pitch cell (B) and the area of a single vessel cell (C) in the stem and xylem of SlHB8 overexpression and SlHB8 knock out lines as well as WT plants. The calculation was performed on Image J softer ware based on the images of toluidine blue-stained anatomical sections as described in the Materials and Methods section. Error bars mean ± SE value. Stars indicate the statistical significance using Student's t-test: * p-value < 0.05, ** p-value < 0.01, *** p-value < 0.001.

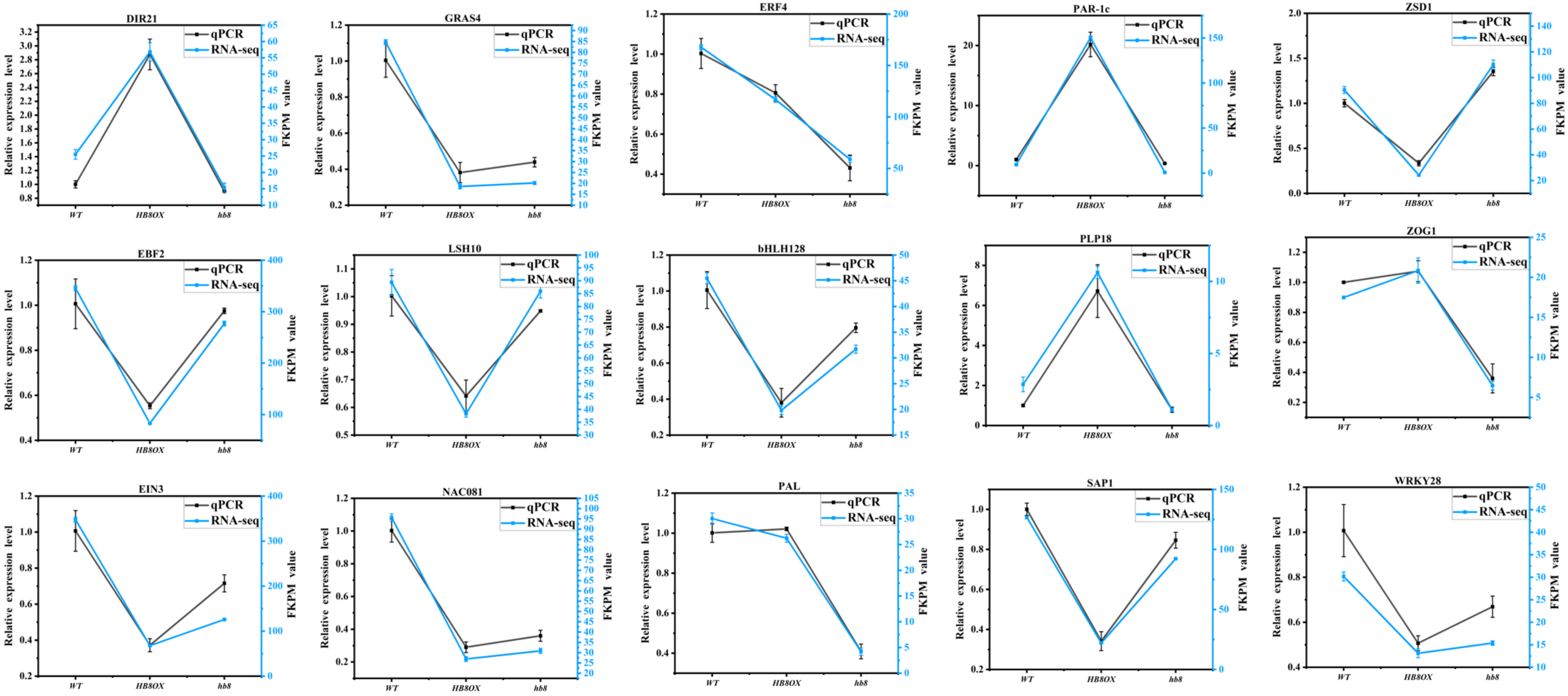

Figure S3. Validation of the expression data from RNA-seq assay by quantitative real-time PCR (qRT-PCR). Relative expression levels of eight genes determined by qRT-PCR (black) and FPKM obtained by RNA-seq (blue). Results for each gene are based on three biological and three technical replicates. Error bars indicate standard error.
