# Supplementary material for: The SlHB8 Acts as a Negative Regulator in Stem Development and Lignin Biosynthesis
Source: Int J Mol Sci. 2021 Dec 12;22(24):13343. doi: 10.3390/ijms222413343 (PMC8708474; doi:10.3390/ijms222413343)
Supplement: Supplementary file 1 [file ijms-22-13343-s001.zip › ijms-1492812-SI/Supplementary Table S1 Summary statistics of RNA-seq data of the 9 libraries mapped to the tomato reference genome (Solanum lycopersicum ITAG4.0).pdf]

Table S1. Summary statistics of RNA-seq data of the 9 libraries mapped to the tomato reference genome (*Solanum lycopersicum* ITAG4.0).

| Samples | WT | | | SlHB8-ox | | | SlHB8-cr | | |
|---|---|---|---|---|---|---|---|---|---|
| Library | WT-1 | WT-2 | WT-3 | HB8-ox-1 | HB8-ox-2 | HB8-ox-3 | SlHB8-cr-1 | SlHB8-cr-2 | SlHB8-cr-3 |
| Clean reads | 50428308 | 49843612 | 53550906 | 49366488 | 45490476 | 39049388 | 43901952 | 63555660 | 49914082 |
| High quality clean reads (%) | 50336792 (99.82%) | 49761328 (99.83%) | 53457896 (99.83%) | 49276912 (99.82%) | 45419064 (99.84%) | 38982904 (99.83%) | 43826050 (99.83%) | 63450750 (99.83%) | 49845336 (99.86%) |
| Removed rRNA reads (%) | 50188458 (99.71%) | 49628866 (99.73%) | 53313056 (99.73%) | 49143478 (99.73%) | 45302194 (99.74%) | 38879150 (99.73%) | 43712426 (99.74%) | 63292294 (99.75%) | 49710888 (99.73%) |
| Mapped reads (%) | 48861307 (97.36%) | 48326637 (97.38%) | 51861046 (97.28%) | 47706778 (97.08%) | 44064712 (97.27%) | 37727049 (97.04%) | 42601874 (97.46%) | 61843685 (97.71%) | 48411175 (97.39%) |
| Unique mapped reads (%) | 47717462 (95.08%) | 47197528 (95.10%) | 50647131 (95.00%) | 46514713 (94.65%) | 42962616 (94.84%) | 36785345 (94.61%) | 41597363 (95.16%) | 60333093 (95.32%) | 47246911 (95.04%) |
| Multiple mapped reads | 1143845 (2.28%) | 1129109 (2.28%) | 1213915 (2.28%) | 1192065 (2.43%) | 1102096 (2.43%) | 941704 (2.42%) | 1004511 (2.30%) | 1510592 (2.39%) | 1164264 (2.34%) |
| All genes | 22546 (64.97%) | 22534 (64.94%) | 22644 (65.25%) | 22568 (65.03%) | 22414 (64.59%) | 22265 (64.16%) | 22447 (64.69%) | 22885 (65.95%) | 22593 (65.11%) |
| Known genes (%) | 21968 (64.47%) | 21961 (64.45%) | 22063 (64.75%) | 21985 (64.52%) | 21831 (64.07%) | 21681 (63.63%) | 21870 (64.18%) | 22307 (65.46%) | 22016 (64.61%) |
| Novel transcripts | 578 | 573 | 581 | 583 | 583 | 584 | 577 | 578 | 577 |
