# Supplementary material for: The SlHB8 Acts as a Negative Regulator in Stem Development and Lignin Biosynthesis
Source: Int J Mol Sci. 2021 Dec 12;22(24):13343. doi: 10.3390/ijms222413343 (PMC8708474; doi:10.3390/ijms222413343)
Supplement: Supplementary file 1 [file ijms-22-13343-s001.zip › ijms-1492812-SI/Supplementary Table S5 List of primer sequences of genes used in the qRT-PCR and vector generation.pdf]

Table S5. List of primer sequences of genes used in the qRT-PCR and vector generation.

Primers for qRT-PCR

	Primers for qRT-PCR	
Gene Name	Forward (5'-3')	Reverse (5'-3')
SlWRKY28 (Solyc01g079260)	CGCCAATCAGTCCTTCAACG	ACGCCTTCCTTGAGCAACAA
SlGRAS4 (Solyc01g100200)	TTGTAGCAGCAGCATTTTGGA	CTGCACGTCAAGTGAGAGAAA
SlPLP18 (Solyc02g093580)	ACGATGACTAGTTGACGATAGTG	AAACGATGAGATATAAGTTTTGCGA
SlSAP1 (Solyc03g007760)	GACTAAACCAGGCGAGCGTA	TCTCAGCTGCAGGTAAACCG
SlPAR-1c (Solyc03g025670)	GGCCCAGTTGATAGCATATAGT	CAATAATAGATGCATTTCTCCACCT
SlPAL (Solyc03g042560)	ATGTTGATGATCCTTGCAGCTC	TCGTCCTCGAAAGCTCCAATC
SlNAC081 (Solyc04g009440)	ATCGACACCAGCAAACGACT	CACATGGCGACAAGACATGC
SlZOG1 (Solyc06g062330)	TCCGTCTACAGGCGGTTTTC	CAGTCATAGGCATAGCTGCCA
SlEBF2 (Solyc07g008250)	TTGTGATGCTCCAGTGGCTT	CGTGTGAAACCGGAGACTGA
SlERF4 (Solyc07g053740)	TGTGTCGGATTCGTCTTCCG	CCATAGGTGGCGCAAGGTTA
SlbHLH128 (Solyc09g098110)	ACTGCTGCAGGTGATACTGTT	GAGCTGAGAGTTCAGCCTTGT
SlLSH10 (Solyc10g008000)	CACCAGCTCCCTGTACTTGT	GCGAATGGGTTGGTCTCACT
SlDIR21 (Solyc10g055200)	TACGTCTCATTCGCCTACGG	TGAGCTCGACCAATAGTCGTT
SlEBF2 (Solyc12g009560)	TTGTGATGCTCCAGTGGCTT	CGTGTGAAACCGGAGACTGA
SlZSD1 (Solyc12g056600)	GTCACTGTCTTACTTTATGTACACG	AACGGTAATGTAATACTCCAAATCG
SlHB8 (Solyc08g066500)	CGTGGAGCTGCTCAAATCTG	CTGGCAGTGCCTTCATAGAG
SlCCR1 (Solyc06g068440)	GCTGGAGCGATCCTGATTTT	ATATGAAGAACACTAGCATTCACAGTATT
SlCYP73A14 (Solyc05g047530)	TTGTGTTCAAGCCTGTTGCTG	GTGTTGTTGTAAAGGCACAGAGT
SlCCR2 (Solyc03g116910)	AGTCTGCAGGAGAAGGGTCA	TTCATAAGCACACGCAGTAGC
SlCCoAOMT5(Solyc10g050160)	GCCCTTGCTATCCCCGAAG	TCAAGAAGGGGCAAAGCAGG
SlC3H(Solyc01g096670)	CAACTTTGTGCCTCAAGTCCAAT	CCAATGTCTTTAACCAACCAGAAAC
SlCAD(Solyc01g107590)	ACCTAAGCAATGTCTTGCCCT	GGAGAATAGTTCAAAGGGAATGGA
SlCOMT(Solyc03g080180)	ACTCCCTTGTAAGAACCCAGATG	CACCATCGGCATTCTTAGTCA
SlHCT(Solyc03g117600)	CTCCCCTTTTCTTAATCCAGGTAAC	CATGGCAAAAGGGTCTGCTA
SlPER3(Solyc05g046020)	TGCTGAATTTGCTCAATCAATGG	ACATATTGCCCCAAACCAAAGA
SlUBI(Solyc01g056940)	CCAAGATCCAGGACAAGGAA	CCAAGATCCAGGACAAGGAA

Primers for Dual Luciferase Assay

	Primers for Dual Luciferase Assay	
Gene Name	Forward (5'-3')	Reverse (5'-3')
ProSlPER3	tatagggcgaattgggtaccGAAGGTCCCCTACCCTTGTAT	tatgtttttggcgtcttccatggTAGGAGCCAGGACAACTCACA
ProSlCYP73A14	tatagggcgaattgggtaccTGGCTCCCTAATCTTGGCAC	tatgtttttggcgtcttccatggTCTTGCTCCTGTTGAGTGGC
SK-SlHB8	cgggctgcaggaattcATGATGGCTGTGACATCAAGCT	cggtatcgataagcttTCAGACAAAAGACCAATTGATAAAC
