# Supplementary material for: The SlHB8 Acts as a Negative Regulator in Stem Development and Lignin Biosynthesis
Source: Int J Mol Sci. 2021 Dec 12;22(24):13343. doi: 10.3390/ijms222413343 (PMC8708474; doi:10.3390/ijms222413343)
Supplement: Supplementary file 1 [file ijms-22-13343-s001.zip › ijms-1492812-SI/Supplementary Table S6 SlHB8 binding sites analysis of the promoters of SlCYP73A14 and SlPER3.pdf]

Table S6. SlHB8 binding sites analysis of the promoters of SlCYP73A14 and SlPER3

3000bp promoter sequence of transcription start site of SlPER3

>SL2.50ch05:58100304..58103303

ACACTTATACTATACTAAGGTCCTATTACCCCCCTAAACTTATTTTATGTGTAATTTTCTACCC
CTTTTTAGCCTACGTGGCACTAGTTCGAAAAAAAAGTCAACCATTGTTGGACCCACAAGAT
AGTGCCACGTAAGCTAAAAAGGGGTAAAAAATTATTAATAAAATAAGTTCAGGGGGGTAAT
AGGACCTTAGTATAGTATAAGTGTGTCTCTGAGATTTCGGGCATAGGTTGAGGGGTACTTGG
GCATTATCCCTATATTAAATTTGATACTTTGAGAAATATATTAGATATCAAATAGTTATATTTAAT
TTCAAAGATAAAATAGAAAAAAAATTATGTCTAAGGTGAATAAGTTTATTGGATAACTATGGT
AATGCTGAACTGCCAGAAAATTTGGCTATACATTGAAAAATTCTATTATATCTTTTTAAATTTT
AAAATAAACTGATCTTTTTTCAACTTTTTGATTAAAAAGTAAAAATGTGCAAAAATAAATAC
AATAACATAGTGTTAAATATATTATCTAATCATTTTGATCAAATTCTAACTAAATTTTTTGACTA
ATTTCTTTTTCCATTTCTAATATAATAAATAAATAAATAAAGATAAATAGAGTATAAAAGTTCT
CCTTTATAAATATATACATAAAAAATATAATAGAAACATGAAGGTCCCCTACCCTTGTATACAA
CAAGGAAAACAAATTCTGGGAACAGTAACCAAATTACTTAATTATGAGTATATGGTCAATGT
CTTATCTGCCAGCAATTGCTTGATACAACCTGTTAATAACTACTTCCTCCCTCTAACTCATTTT
TTCATTTTTTTAAAATCAAATTGATAATCATTAAAACTTAATTGAATCAGATCAATTCAACATT
TTAAAATTTTGATAATTAAAACTATACTAAAAGTCCATATTAAAACTATACTAAAAGTCCATA
AGTCCATGTTCAATTTTTTTTTGAAAATTAAACAGTTTTTCCGCATTTTTTTATTAAAATCAAA
TCTCAATTATGACTCAAATCTAATGTATATCCTTACATTATCTAAGTGAACTTAAAACATCCTT
ATATTATCCAAAATATAACAAGAATCAACCTTCAGTCTATTTTTGTTAAAAAACTAATTGAAC
CAATAAAAATAATAATTTGATCATGTGTTAGCAACAAAAGAGAAGATACAAGTACCTTCAAA
AAAAAAAGTACCTTAAAAAATATGGATATGAATTGAAAATGATTATTTCATTGTCATAATTTA
ATTTTTTTGCGTTTTTAATTTATATGTTTTTTGCATCATCAAGTAAATTGACTTGCAACAATAC
ATAAGTTTTGAAATATTTATATATTAGATTACGATGATTAGAGAGATTATGTAAAAAAAAATTA
TTTTAAAAATATTATGTTCAACTAATTAGAGACTTTCTTTAATAAAATATTTAGTATGTGTACTA
TATGTATATAATAAAATTCACATTTTATACAATGTAAAAAAATACTGAAAATTTAGTGAAATTA
CGTAGGCATTTGGTTATGAAAATTAAAAAATAATAATTACTTTTGTTTGAAAATTTTAAAGTT
GGAGCTGTATTTGGCTATACTTTTTACAAAGAATATTCAGAATTTGAATATATTTTGTCTTAAT
ATGATTTAAACTTTCAATTTCTAAAAGTTCGACTTGACACATTTTTTTGATTGCTTGAGCTTT
TAAAAAAAATAGAGAGAAGGATTGCAACTTTTTGAATAATTTAAGGATATTTTTTGTTCAATT
ATGTAACACAAGGATGTACTTTAAATTTATGTCATACTTCAAGAATGATTTTAGCCAAAAACT
CTAATTTGTACAATTTATATGAAACCTTAATCTAACTGTTTCAATTTTGAGAAAACGTCAAAC
AAGTAGCAAAGTAATAATGAATTGATGTGGACATATGTCAAAAGCATATTTGCATATGAGAAT
ATATGTGAGTTGTCCTGGCTCCTACATATAATTTCTGATACAACTTGAGGAGCAGGTGAGTTT
TTCTATTTTTCAATTCCAATAATACCAACCATAAAAGCACAAAACAATTAAACTGATCAAGA
AAATGATAAAACACAAAGATTTCGTTTATTTATGTGTGCGAAAAACAAATAAGTAATATATAT
ATATATATTTTTTGGAGAGAGAGTTAAATATATATCTAAGAATTCACGTAAAACATCTAAATTC
AGGATTAATCATGTTACATATGTATATGGGGAAAAAATACAAGTCTTTTGTTGTTGTTGGTAA
AATGGAAAAATAAAAAATTAATAAAAGTTCTTAAAGTGTCCATAAACAACCGGACTTAAATC
TACTAAAACAAATTTTATGTCATTTATTAAGTGCATTCATTGTAACAAAATTAACTTTTAGGG
GTAATAATATGCATATTAAAAAAGAGTAAAATATTTATCGCATTAGTGTATCATTGGACGCAAT
ATCCTTATTAGCTTTAGAGATATTTACAAAAACTGTTAATTACGTCTACAAAAATATACAGTG

ATAATTAAGTTGTTACCATTGACGACTTATTATCGCTAACGATCACAAGTAATTATAGAGTATT
AGAGACTCTTCATATTTGTAGCTATCATTTACTAGAAAATCGACACACTTCGGACCTGTTTGG
ATTGACTTTTGACTTGTGACTTATAAGCCACAAATCATAAATTAGAATTTTTAACTTATGACT
TTTGACTTATTTTTTTTATTTTGGCTTTAAAATAATTGTTAATAAGCATTTTTTAACTTATGCAA
ACATTTCAAAACTGCTTAAAAGTTATTTTAGCTTAAAAGCACTTAAAATAAGTCAATCCAAA
CGGACTTAATCGTAACCTTATTTAAAATGAAGGATACTAGTGGGAGTATTTATGCATGGAAG
AGGGAAGATGGCCAAACAAAATATGAATATTTATTACCGAAAGAGTACGACAGACTAACTAT
TTAATTAATCAATCAACCAAACAAACAA

3000bp promoter sequence of transcription start site of SlCYP73A14

>SL2.50ch05:59020065..59015066

ACAAATTTAGATATGTAAATGTATGTTGGAAAAATATATGGATTAATACTTTTTAATAAGTAAT
TACTGATTTTAGCGACTTTTTCTTTAGGTTAAATTCATGGGTGGCTCCCTAATCTTGGCACCA
ATTTTCACTTAAGTTCCTCAATTAGGCAATTTTCATTTTAGACACCTCATGTAGGGTTCTATTT
TGTCATTTTGACATTTTTCTAACAATAAATAAAAAACTAAAGGAGAGTGTAATACGCTTGTT
GATGACGTGACAAACAATCAATTAAATAATTATGTGTGTTATTTTCAATTAAAAATAATTATAA
AAATCTATTTAGTAAATAAATTAAAATATTATTCTAAGAAAATCTCTATTTCATTACACTCAAA
ATCTATTTTGTGTTTTCTCAAAAAAAATATTTTTTCTTTTACTATTTTTCATATTTATTCATCTT
CTTTCTTTTTAGTATTGTTTTTGGAAAGAAAAGAGGGAGAGGAAGAAAGAGAAGAGAATA
AATATGAAATATGGGTGTTGGTATCCATTTTTTTGGCAAAATATGGAGGAATGTGATGATAGA
TTTAGATAAGTAATACGATGAAGAAGAAGAAAATGACTTAAAAATGAACTTGAATAAAAAA
TTTGACCTCCATTGAAGGAATTTAAGCTTGAAAAAAGGATAGGGGGTGGTGGCTTGAAAAA
TGGAGAAGAAGAAGAGAAAATAAAATTAAAAATGGCTAAATTAAGTCTTTCACGCGATATT
GTTGAGTATATCTCACTCACTTTGACATGTTAGCGAAAAATGTCAAAATGATACAACAGAAC
CCTACATGAGGTGTCTAAAATGAACATCGCCTAATTGAGGTGTTTAAGTGAAAATTGGTGCC
AAATTTAGGGGGTCACCGATAGATTTGGCCTTTTATTTATCACCATTTATAGTAACACATAAC
AATACTATGTTAAATCTGCAATATGTATTAAAAGTAATTTATGTATGCAATATATATGTATTATA
ACTGTTTTTTAAACTATATTATGCTTGTTTGAAAAGAAATTGACATATTGTATTATATGAGTATT
AAAATGTGTGATAAATGTATTAACCATCAATAACGCTACTATATGTGAATAACATATTCTTCTT
TGTAATATACATTAAAATTGTATAATAAATGAACTAGTAAAAATACCCGCGTTTCGTGCGGGA
GTTAAATATTACAAAATTTATAAAATAATACTTAAAGTACATCGGTCATTAAACCTAAAATTCT
ATATTCTCTTACACACAAGATTATGTAGTAAGAATCATTAACGATGTAAAGGAACATAAAAAT
TATTGCATCTTATCATTTATCCTTAATAATATAAGCATAAACTAATGAGTGTAACAATACATACC
AGGATCTGTGAGATAAACAATGATGCTTGTTAGCCACTCAACAGGAGCAAGATACATAAATA
TTTAAGAAGAAGAAGTTTAAAATTTTTATTTTTTTTAAAATGGGAGGAAAACTCACTATTTAT
TGTGTGAACAAAGGGTAGTGTGGAAGAATATTTATTGTGGCTTATCGAAAAGTTACAACTAA
TTGGAAAAGTTACAATCCTTCATTAATGTCACAATCTTTCAGAAAAATCACAACTTTTCATA
AGAATCGCAACTCTTTATTAAATCACAACTTTTCATAAAAGTCGCAATTCTTTATAAAAATCG
CAACTCTTCATTAATGTCGCGATTTTTTATAAAAGTCACAACATCCATAAATATCACAACTTT
TCATGAAAGGTGAAGGTTAGTTTTGTAAATAAATTAATTAAAAGAGAATTCTTGTTTGTGGT
GGCGCCACGTAGGCGTGCCTAAGATTCTCTTTTATATAATTATATGATATGATTAAAAATGATC
AGGTGAAAAAAATATTATTGTTATAACTGATAAATATTTTTATAATGTAATATATTTATGTAAGT
TTCCCGAAACCTATACATTTTGGGTTTTATATAATGAATTGATGATGAATTTGTTTGGTTATAC
CCAAAGAATTAGACATAAGAATATATGTGGTATAATGTTTTAACTTCTACTTGTTTTAAAAAAT
ATTAAAAATTAATCGATTTGTATCAATGTTAAAAAGAAACCAAGATAATGAATTGGTTTCAA
GAAAAAAAAATTAGTTATACAAATCAATTAATAAAAATATTAATTTAAGATAAATTTTATAAA
ATAACTACGTAACAATATCATTGACACCTTAATTACTTTTCGTTATAATCTAAATAATTTTTGT
GAATATATGGAATCGTATTTATCACTCGAATTAGGCTAGTAGATATGTCATAATACCAAATATT
TGTTGATTACATTTAGTAATCTTTATCATGTTATGTATTAATGTTTATTATTAAAAAAAACTATC
TATAATTACTCAAAATTGACCTTATTATATAGAATTCAACTCTTGCTTTTTATATTAGTGGCAG
CCATCTTAAAATGTGGAAACTTTAAAATGGAATTAGTTCAAGACCAATTGGATTCTTTAATCT
CTCATTGGACATAAATTTTGAAGTAGTCGTATTTGAACATCCGTTTTACTTAGAAAAAAATTG
AAATTATGTGAATAAAAGTAAAATTTCTCCAAATAAGGTTTTGAAAAAAATAAAAATATTCG

AAGATCAAATATTCAAAATCTAAAAAAAAATCAAATGATTCATGGCTAAATTAATATTTAAAAA
TAACTTTTTAAAATTTGAATTTACGATCAAACGATAGCTTACCTATGACATTAAGATTACGTA
GAAAAAATGTTTAAATGTAGATGAATGACATTTTTGGCTTTACCAAACATGAATCTACTAGTT
TTGACTTGATTAATAGATAGCCACTATATACATTCTCCTACCTAGAATTTGAACATGCCCATGG
ATTATACACAAAACCATTTGACAACAAACTTTCCTACCCCCACCATGTTGTCAATTTTACTTC
ATATATATACACATAATTAACACCTTATGC
